# Molecular Control of Sporophyte-Gametophyte Ontogeny and Transition in Plants

**DOI:** 10.3389/fpls.2021.789789

**Published:** 2022-01-13

**Authors:** Saurabh Pandey, Amir Bahram Moradi, Oleksandr Dovzhenko, Alisher Touraev, Klaus Palme, Ralf Welsch

**Affiliations:** ^1^Faculty of Biology, Institute of Biology II, Albert-Ludwigs-University of Freiburg, Freiburg, Germany; ^2^ScreenSYS GmbH, Freiburg, Germany; ^3^National Center for Knowledge and Innovation in Agriculture, Ministry of Agriculture of the Republic of Uzbekistan, Tashkent, Uzbekistan; ^4^BIOSS Center for Biological Signaling Studies, Albert-Ludwigs-University of Freiburg, Freiburg, Germany

**Keywords:** ontogeny, sporophyte, gametophyte, alternation of generations, phase transition

## Abstract

Alternation of generations between a sporophytic and gametophytic developmental stage is a feature common to all land plants. This review will discuss the evolutionary origins of these two developmental programs from unicellular eukaryotic progenitors establishing the ability to switch between haploid and diploid states. We will compare the various genetic factors that regulate this switch and highlight the mechanisms which are involved in maintaining the separation of sporophytic and gametophytic developmental programs. While haploid and diploid stages were morphologically similar at early evolutionary stages, largely different gametophyte and sporophyte developments prevail in land plants and finally allowed the development of pollen as the male gametes with specialized structures providing desiccation tolerance and allowing long-distance dispersal. Moreover, plant gametes can be reprogrammed to execute the sporophytic development prior to the formation of the diploid stage achieved with the fusion of gametes and thus initially maintain the haploid stage. Upon diploidization, doubled haploids can be generated which accelerate modern plant breeding as homozygous plants are obtained within one generation. Thus, knowledge of the major signaling pathways governing this dual ontogeny in land plants is not only required for basic research but also for biotechnological applications to develop novel breeding methods accelerating trait development.

## Alternation of Generation – Definition and Common Themes

The life cycle of land plants alternates between two generations: a diploid sporophyte and a haploid gametophyte, with each generation developing a multicellular body. The concept of alternation of generations was first proposed by the German botanist Hofmeister (1851). Hofmeister (1851) termed these fundamental phase transitions with the German word *Generationswechsel* which is still used to specifically describe the process (Horst and Reski, 2016). With the advance of molecular techniques and knowledge, these morphological observations have accumulated the molecular support that allows us to precisely define and understand the *Generationswechsel*. At the molecular level, a single plant genome encodes two fundamentally different programs, governing the development of two different body plans (ontogenies; Horst and Reski, 2016). The gametophytic generation represents the haploid phase of the plant’s life cycle during which gametes are produced by mitotic division of haploid spores, whereas the sporophytic generation represents the spore-producing diploid generation (Friedman, 2013). In land plants, both haploid and diploid cells can divide by mitosis leading to the formation of different multicellular haploid and diploid plant bodies (Bowman et al., 2016). The haploid plant body representing the gametophyte produces gametes by mitosis which after fertilization form the diploid zygote. Following mitotic divisions, the zygote produces the sporophytic plant body. Depending on the relative period of the developmental process that each phase occupies, either the gametophyte or the sporophyte is considered the dominant stage in the respective plant species (Bowman et al., 2016). In mosses, the haploid gametophyte generation is dominant, whereas in vascular plants (ferns, gymnosperms, and angiosperms), the diploid sporophyte is the prevalent generation. Fertilization, the fusion of two haploid gametes to a diploid sporophyte and the generation of haploid gametophytes from a diploid sporophyte through meiosis, are two processes that act as switching points for haploid-to-diploid and diploid-to-haploid transitions, respectively (Horst and Reski, 2016).

Remarkably, a single genome governs the two generations or ontogenies as well as encodes the regulatory mechanisms to switch from one to the other (Friedman, 2013). Improper phase transition can have severe consequences for any of the plant species including loss of the capability of sexual reproduction (see below). Thus, this transition must be under tight molecular control including key regulatory genes which initiate phase transitions and govern distinct developmental processes occurring in the gametophyte and the sporophyte. Obviously, regarding the fundamentally different programs as well as the resetting of cellular identities with the switch from one program to another, epigenetic control mechanisms are similarly involved. Some of the genes known to be involved in ontogeny determination and phase transitions are discussed in the following section while epigenetic control mechanisms from haploid-to-diploid switch are covered by other excellent review articles (Borg et al., 2021; Ono and Kinoshita, 2021; Vigneau and Borg, 2021). Moreover, the focus of this review is on mechanisms of diploid-to-haploid and haploid-to-diploid switches in angiosperms as a requirement for the development of novel biotechnological breeding approaches.

**FIGURE 1:**
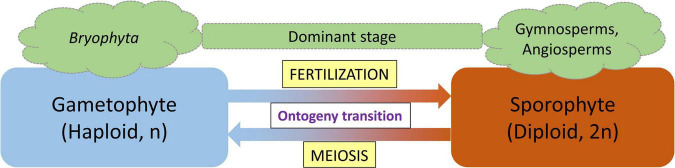
Alternation of generation life cycle pattern of land plants. Lower plants (*Bryophyta*) spend the majority of their life in the gametophyte stage, whereas the sporophyte stage is the dominant stage in vascular plants (gymnosperms and angiosperms). Fertilization and meiosis function as ontogeny switch points.

The complexity of multicellular flowering plants has its origins in relatively simple early land plants (Figure 1). *Phaeophyta* (brown algae) evolved 150–200 million years ago and colonize mostly marine environments, and *Sargassum*, *Ascophyllum*, *Fucus*, and *Ectocarpus* are some of the best-known members of this phylum. Their evolution parallels that of the green algae and red algae as all three groups possess complex multicellular species with an alternation of generations. With the origin of a phase transition, the gametophytic and the sporophytic generations were morphologically indistinguishable (isomorphic) while during evolution distinct developmental programs were accompanied with partially large morphological differences between the two generations. Accordingly, members of *Phaeophyta* show various types of alternation of generation, i.e., isomorphic (*Ectocarpus*) or heteromorphic (*Laminaria*; Bringloe et al., 2020).

Species of *Chlorophyta* (green algae) are common inhabitants of marine, freshwater and terrestrial environments with a simple body plan and *Chlamydomonas* is one of the most studied members of this phylum. All land plants (*Embryophyta*) are believed to have evolved from *Chlorophyta* and feature a progressive increase in complexity with the evolution of bryophytes, pteridophytes, gymnosperms to angiosperms (flowering plants). The progenitors of early land plants (*Chlorophyta* and *Charophyta*) developed multicellularity, but do not have dual ontogenies (Finet et al., 2010). Dual ontogeny came into existence in land plants only after the appearance of *Bryophyta* and it remained in all the land plants thereafter (Jill Harrison, 2017).

The coordination of reprogramming events at the molecular/genetic level defines the integrity of ontogenic decisions (Hafidh and Honys, 2021). Here, developmental decisions mediated by members of the three-amino-acid-loop-extension (TALE) class of homeoproteins characterized by a highly conserved DNA-binding homeodomain (HD), are prominent (Bowman et al., 2016). The most important HD proteins belong to the families of KNOTTED1-like (KNOX) and BEL1-like (BLH or BELL) which function as heterodimers (Arnaud and Pautot, 2014).

In bryophytes, the gametophytic phase dominates the plants’ lifecycle, whereas the sporophytic phase is very short. In contrast, the sporophyte phase became dominant in vascular plants (gymnosperms and angiosperms), however, the evolutionary pressure which caused this development is still an open and intriguing question for evolutionary biologists. Possible investigations could focus on finding the genetic factors that control following aspects:

• Molecular mechanism of the phase transition.
• The maintenance of specific body plans.
• The timing of transitions between one body plan to another.

The pervasive influence of ontogeny control exerts a strong evolutionary pressure that would be expected to result in the evolution of rigid checkpoints and the clear separation of the two developmental programs. Initial elucidation of the genetic control of gametophyte-sporophyte ontogeny determination and phase transition in different plant species has already provided strong evidence for a common genetic program controlling all haploid-to-diploid transitions. Homeodomain proteins play a central role in this checkpoint (Bowman et al., 2016). Here, we review our understanding of some of these molecular controls in different taxa.

## Alternation of Generations in Algae

### Brown Algae (*Phaeophyta*)

The majority of brown algae (*Phaeophyta*) exhibit an alternation of generations in which either the gametophyte or sporophyte can be the dominant stage. *Ectocarpus* is a filamentous brown alga that is used as a model organism to study the life cycle and developmental events (Coelho et al., 2012). Life cycle mutants *ouroboros* (*oro*) and *immediate upright* (*imm*) were identified in *Ectocarpus* and provide molecular information about life cycle progression in brown algae. *Oro* acts as a single, recessive Mendelian locus that is unlinked to the locus of *imm* mutant and is crucial for sporophyte development. ORO is considered to be a master regulator of the gametophyte-to-sporophyte life cycle transition (Coelho et al., 2011), whereas IMM is required to induce the partial conversion of sporophytic to gametophytic generations (Peters et al., 2008). Transcriptome analysis shows that ORO induces the sporophyte developmental program and represses the gametophyte genetic program (Coelho et al., 2011). More recently, SAMSARA (SAM) has been identified as an interacting partner of ORO (Arun et al., 2019). SAM and ORO form a heterodimer that regulates the expression of genes controlling gametophyte to sporophyte generation, mainly associated with functional categories like “Cell wall and extracellular” and “Cellular regulation and signaling” (Arun et al., 2019). This suggests that TALE-HD transcription factors are of ancient origin and function as gene regulators during sporophytic developmental events.

### Green Algae (*Chlorophyta*)

*Chlamydomonas reinhardtii*, a unicellular green alga generates two types of gametes, plus-gametes and minus-gametes. The fusion of the gametes, the haploid-to-diploid transition, is regulated by many factors, including GAMETE-SPECIFIC MINUS1 (GSM1)/GAMETE-SPECIFIC PLUS1 (GSP1) which form a heterodimeric transcription factor (Table 1).

**TABLE 1:** Gamete-type specific proteins in *Chlamydomonas*.

Gamete type	Expressed protein	Protein family
+	Gamete-specific plus1 (Gsp1)	BELL-related TALE homeodomain protein
−	Gamete-specific minus1 (Gsm1)	KNOX-related TALE homeodomain protein
Zygote+ −	GSM1 + GSP1	

The plus-gametes express *Gsp1* which encodes a BELL-related TALE-HD protein whereas the minus-gametes express *Gsm1* encoding a KNOX–related TALE-HD protein (Lee et al., 2008). Interestingly, these proteins are structural and functional homologs to KNOX/BELL homeobox heterodimers which are engaged in the sporophytic, diploid phase in vascular plants (Lee et al., 2008). During nitrogen starvation in *Chlamydomonas*, the vegetative cells differentiate into plus- and minus-gametes which display SAG1 (sexual agglutination) and SAD1 (sexual adhesion) agglutinins on their flagella membranes, respectively. The agglutinins on their flagella surface cause adhesion between complementary gametes leading to initial recognition events, triggering an intracellular cAMP burst that activates gametolysin, an enzyme that degrades the cell wall to allow membrane fusion between two gametes. This takes place between plus and minus mating loci structures expressing membrane fusion-enabling factors such as FUS1 and HAP2 on the plasma membrane, respectively (Liu et al., 2010). The *fus1* gene encodes a glycoprotein that enables fusion and binding to minus-gametes, while HAP2 plays an essential role in the completion of the membrane fusion process. The species-specific adhesion initiates the fusogenic reorganization of HAP2 from a labile form into a stable homotrimeric form. Hydrophobic residues of the HAP2 homotrimer subsequently interact with lipid bilayers and are involved in converting them into a single lipid bilayer as the fusion product. The adhesion thus exhibits two functions: it allows sex cells to recognize each other, and initiates the biochemical conformational changes required to activate the fusion machinery (Zhang et al., 2021). Following cytoplasmic fusion, FUS1 and HAP2 are degraded (Liu et al., 2010), and the two HD proteins GSP1 and GSM1 physically interact to form a heterodimer and translocate from the cytosol to the nucleus, initiating the zygote developmental program (Liu et al., 2010).

The ectopic expression of GSP1 in minus-gametes results in the transcription of certain genes that would otherwise be exclusively transcribed in zygotes. Lee et al. (2008) extended these findings and reported the molecular regulation of haploid-to-diploid transition through the KNOX-TALE genes in *Chlamydomonas reinhardtii*. Moreover, the ectopic expression of these proteins in vegetative cells is sufficient to activate the zygote development. A broader comparative analysis led to *KNOX-TALE* genes in land plants being proposed as candidates for the regulation of alternation of generations (Lee et al., 2008).

## Alternation of Generation in *Bryophyta* and *Lycophyta*

Land plants comprise bryophytes, lycophytes, ferns, gymnosperms, and angiosperms. There is a good amount of evidence to highlight the conservation and evolution of ontogeny control and determination in these land plants as described below.

### Bryophyta

Bryophytes spend the majority of their life cycle as persistent haploid gametophytes and exhibit only a short-lived diploid sporophyte generation (Cove, 2005; Szövényi et al., 2021). Bryophytes include three lineages, namely hornworts, liverworts, and mosses.

#### Hornworts

Hornworts are a small clade consisting of about 220 species with the majority being present in tropical regions. *Anthoceros agrestis* is the model plant species for this clade whose genome was recently sequenced (Li et al., 2020; Frangedakis et al., 2021). Hornworts share some common features that connect them with both the green algae and other land plant lineages. Similar to green algae, they have a single chloroplast per cell with a characteristic pyrenoid which is functionally associated with carbon-concentrating mechanisms. Similar to vascular plants, the sporophyte of hornworts is long-lived and develops moderately independent from the gametophyte which represents the dominant stage (Li et al., 2020). While a KNOX1 ortholog is absent in the *Anthoceros* genome, several *KNOX2* genes are present. In *A*. *agrestis* a single BELL and a single KNOX2 gene are specifically expressed in the sporophyte phase (Li et al., 2020). As BELL is expressed during early stages of sporophyte development while KNOX2 shows the opposite pattern with expression during later stages, sporophyte identity might not be determined by KNOX2/BELL interaction. However, detailed functional reports are presently lacking which would be instrumental to understand the involvement of KNOX and BELL genes in zygote activation and ontogeny control in hornworts.

#### Liverworts

In contrast to hornworts, liverworts or *Marchantiophyta* are a larger clade with an estimated number of 9,000 species (Christenhusz and Byng, 2016). Like all *Bryophyta* they are gametophyte-dominant. *Marchantia polymorpha* is the model system of liverworts with its genome sequenced in 2017 (Bowman et al., 2017). Analysis of its genome for HD-containing genes involved in haploid-to-diploid transition revealed four *KNOX* genes and five *BELL* genes. Among the *KNOX* genes, three belong to the KNOX1 subclass, however, only one gene, *MpKNOX1*, encodes a HD protein while the remaining two (MpKNOX1A and MpKNOX1B) lack a HD. *MpKNOX1* is expressed specifically in developing and mature egg cells and is absent in the male gametophyte. In contrast, the forth *Marchantia polymorpha KNOX* gene, *MpKNOX2* is not detected in unfertilized reproductive organs and expressed primarily during sporophyte development (Dierschke et al., 2021; Hisanaga et al., 2021). Thus, *MpKNOX1* is the only *KNOX* gene in *Marchantia polymorpha* involved in phase transition regulation.

Among the five *BELL* genes, *MpBELL1* is expressed primarily during sporophyte development similar to *MpKNOX2*, while *MpBELL5* is expressed in archegonia and functionally not characterized. In contrast, the remaining three *BELL* genes, *MpBELL2*, *MpBELL3* and *MpBELL4* are expressed in the antheridia in cells which will develop into sperm cells (Dierschke et al., 2021; Hisanaga et al., 2021). Upon fertilization, egg-derived MpKNOX1 and sperm-derived MpBELL3/4 heterodimerize and activate the transcription of zygote-specific genes, which is essentially required for diploid sporophyte development. Confusingly, however, *MpBELL2*,*3*,*4* expression is not exclusive for sperm cell, alternative short transcripts of *MpBELL3* and *MpBELL4* are also detected in egg cells. However, truncated MpBELL3 and MpBELL4 proteins are incapable to interact efficiently with MpKNOX1 in split YFP BiFC assays in a heterologous system, which is considered as one reason that sperm-derived full-length MpBELL3 and MpBELL4 are required for KNOX/BELL heterodimer formation. One hypothetic function of maternal MpBELL3/4 presence is a backup function to ensure diploid development following fertilization (Dierschke et al., 2021).

Interestingly, ectopic expression of either *MpBELL3* or co-expression of *MpKNOX1* and *MpBELL3* in the vegetative gametophyte for 72 h is sufficient to activate both *MpKNOX2* and *MpBELL1*, whose expression is normally limited to sporophyte development. Thus, MpBELL3 alone controls *MpKNOX2* expression, which is reminiscent of the post-zygotic activation of MpKNOX2 after fertilization (Dierschke et al., 2021).

In summary the zygote-activating function of KNOX/BELL is conserved between *C*. *reinhardtii* and *M*. *polymorpha* (Dierschke et al., 2021; Hisanaga et al., 2021). This striking conservation of KNOX/BELL functions in the promotion of karyogamy across phylogenetically distant *M*. *polymorpha* and *C*. *reinhardtii* suggests that functions of KNOX/BELL heterodimers shifted from zygote activation to sporophyte development as land plants evolved (Hisanaga et al., 2021).

#### Mosses

The two ontogenies can be changed easily by modulating the culture conditions making them an ideal system to understand the molecular controls of sporophyte-gametophyte determination and transition (Horst and Reski, 2016). *Physcomitrium patens* is a widely-used model system that has been used to understand the ontogenic switch due to its small genome, non-redundant gene structure, short life span, and the simple mechanical induction of two types of asexual reproduction processes: apogamy (haploid) and apospory (diploid; Cove, 2005).

Apospory can be induced by mechanically injuring sporophytic vegetative tissue (Pringsheim, 1876; Horst and Reski, 2016), whereas apogamy occurs spontaneously in old cultures of several moss species (Bauer, 1959). Interestingly, this feature is lost in isolated apogamous sporophytes. Bauer (1959) suggested that a mobile self-replicating “sporogonial factor” is produced in moss sporophytes that can induce the development of further sporophytes from gametophytic cells (Horst and Reski, 2016). Based on this hypothesis, Ripetsky (1985) postulated that the hypothetical sporogonial factor is under epigenetic control and is switched on for sporophyte development and also observed that a gametophyte moss culture can be established via aposporous regeneration of sporophytic cells. These diploid gametophytes would continuously give rise to apogamous sporophytes, even in the absence of previously established sporophytes.

In the past decade, these observations have received molecular support. In the moss *Physcomitrium patens*, it has been shown that the two transcription factors of the KNOTTED1-LIKE HOMEOBOX (KNOX2) class MKN1 and MKN6 are necessary for maintaining the sporophyte developmental program. Accordingly, *mkn1* and *mkn6* double knockout show gametophyte morphological features in diploid sporophytes (Sakakibara et al., 2013).

In another recent report in *Physcomitrium patens*, it has been shown that the ectopic overexpression of the homeobox gene *BELL1* (*PpBELL1*) in specific gametophytic cells induces embryo formation and subsequent development of reproductive diploid sporophytes without undergoing fertilization (Horst et al., 2016). This demonstrates that PpBELL1 represents the central molecular trigger for gametophyte-to-sporophyte transitions in *P*. *patens* (Horst et al., 2016). Similar sporophytic features in the haploid gametophytic stage are observed in *P*. *patens* mutants of the Polycomb repressive complex 2 (PRC2), which is involved in the control of epigenetic memory (Okano et al., 2009). Moreover, as PRC2 represses BELL1 function in *P*. *patens* and PRC2 homologs are present also in *Chlamydomonas*, it is appealing to envision a PRC2-mediated control of phase transition before the emergence of land plants (Schubert, 2019).

### Lycophyta

*Lycophyta* are one of the oldest lineages of extant vascular plants. Similar to other vascular plants lycophytes reproduce by spores and the sporophyte generation is dominant in these plants. *Lycophytes* consist of three families namely: *Lycopodiaceae* (club mosses), *Isoeteaceae* (quillworts) and *Selginellaceae*. *Lycopodiaceae* members produce one single type of spores (homosporous), whereas *Isoeteaceae* and *Selginellaceae* species are heterosporous and produce megaspores and microspores. The distinct differentiation of male and female sporophytes with largely different sizes and properties in seed plants is considered to be originated in these species, although heterospory is thought to have evolved independently in several plant groups. Living lycophytes represent a sister group to the seed plant clade and diverged from a common ancestor around 420 million years ago. Thus, they are frequently exploited for comparative studies regarding conservative traits and convergent evolution of traits which have evolved independently, such as leaves and roots.

*Selaginella* is the model genus for detailed studies of these plants. *Selaginella* is of particular interest as it retained an autonomous but water-dependent gametophyte generation that is typical of all non-seed plants. In contrast to angiosperms, their gametophytes are not buried within maternal tissues of the sporophyte, so it offers a useful experimental system for investigating how the alternation of generations is regulated. Free-living gametophytes also make it a suitable system to study gametogenesis, gamete recognition, fertilization, and early embryonic developments. Identification of genes involved in the ontogeny control and transition in these plants would help to investigate the evolution of genes and their speciation (Banks, 2009).

Compared to the primary expansion of the number of *KNOX* genes during the evolution from algae to moss, a second expansion occurred during the transition from lycophytes to angiosperms (Gao et al., 2015). While lycophytes contain four *KNOX* genes, angiosperms contain a much higher number which is also interpreted in the context of the development of complex leaves with many specialized tissues leading to a neofunctionalization of several *KNOX* gene duplications during angiosperm evolution.

## Alternation of Generation in Angiosperms

In Angiosperms, the development of the male gametes from microsporocytes or pollen mother cells occurs within anthers, finally forming fertile male gametes - pollen. Angiosperm anthers usually consist of four layers that nourish and protect developing male gametes: epidermis, endothecium, middle layer, and tapetum. The innermost layer, the tapetum, contains sporophytic helper cells that control the development of microspores into pollen but die soon after the first pollen mitosis. The dynamic crosstalk between the reproductive cells and somatic helper cells happens at multiple levels throughout the gamete development. Any defect in this crosstalk leads to non-viable pollen grains which highlight the complexity of the relationship and significance of this developmental process (Feng et al., 2013).

### Diploid-to-Haploid Switch

The main functions of meiosis are the reduction of chromosome numbers and recombination which provides genetic variability. However, it also represents a critical stage for the ontogeny switch, representing the sporophyte-to-gametophyte transition (Hafidh and Honys, 2021). Our efforts to understand meiotic gene functions during the transition from the sporophyte cell lineage (the pollen mother cell) to the gametophyte cell lineage (microspore) are still in their infancy. In contrast, the molecular control of tapetum development has been extensively studied. Tapetal cells never change their ontogeny but instead undergo programmed cell death after performing their specific functions (Ma, 2005; Parish and Li, 2010). Unlike the tapetum cells, the scenario is very different for the pollen mother cells, which after the completion of meiosis have to alter their physiology to accommodate the new ontogeny of gametophyte development (Hafidh and Honys, 2021). Extensive chromatin changes are expected during this transition, with genes involved in this phase transition forming the keys to our understanding of the molecular events that govern the two ontogenies (Hafidh and Honys, 2021).

**FIGURE 2:**
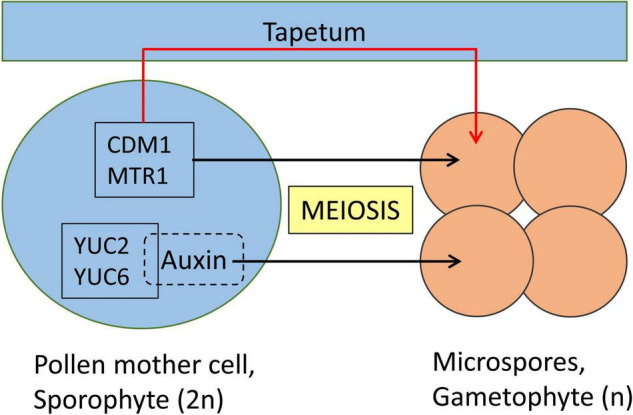
Meiotic genes controlling the gametophyte development in Angiosperms. Pollen mother cell (PMC) and tapetum are sporophyte stages. A diploid PMC (2n) undergoes meiosis to produce four haploid (n) microspores, which are gametophytes. Among the genes expressed in PMC, the function of certain genes is in the development of microspores, thus the PMC development remains unaffected in corresponding knockout mutant lines. CDM1 (in *Arabidopsis thaliana*) and MTR1 (in *Oryza sativa*) are essential for male gamete development which occurs indirectly through the tapetum. Auxin produced by YUC2 and YUC6 plays a direct essential role for the development of microspores. Sporophyte and gametophyte developmental stages are represented in blue and orange, respectively. Direct role of meiotic factors is represented with black arrows, whereas indirect role is represented with a red arrow.

Rice MICROSPORE AND TAPETUM REGULATOR1 (MTR1) is a fascilin glycoprotein that is essential for the development of male gametes (Figure 2). *MTR1* is absent in the tapetum but is expressed from early meiotic (stage 7), tetrad (stage 8) stages until microspore development (stage 9; Tan et al., 2012). Interestingly, even though MTR1 is present at the sporophyte stage of wild-type male reproductive cells, *mtr1* plants show no defects at the meiotic and tetrad stages, but fail to undergo mitosis 1 and 2 and are, thus, male sterile. The programmed cell death of tapetum cells, which is their final developmental stage in normal pollen development, is delayed in *mtr1* mutants resulting in defective sporopollenin deposition which in turn detaches microspores from their tapetal inner surface. This indicates a crosstalk between MTR1 and tapetal cells, which is suggested to involve a secretion of MTR1 during early microspore development regulating tapetum development via interaction with surface proteins. In conclusion, MTR1 serves as a critical signaling protein that coordinates the development of microspore and tapetal cells (Tan et al., 2012; Feng et al., 2013).

Further cases of male sterility were recently observed in crosses made with pigeon pea (*Cajanus cajan*) with its wild relative *Cajanus sericeus* (Pazhamala et al., 2020). One of the lines generated was the thermosensitive male sterile line *Evs Sel 107* in which pollen mother cells undergo normal meiosis and form normal tetrads, however, microspores fail to separate and eventually die. The male-sterile condition of this mutant could be reversed to fertility by reducing the day temperature below the critical threshold temperature of 24°C. The morphological studies were compared with transcriptomic, proteomic and metabolomic experiments revealing that the male sterility was caused by a perturbation of auxin homeostasis (Pazhamala et al., 2020). Confirmatory, external application of a natural auxin, indole-3-acetic acid (IAA), rescued the sterile phenotype emphasizing the critical role of auxin in gametophyte development.

Further support for the involvement of auxins in pollen development has also been reported in *Arabidopsis*. Here, the expression of two auxin biosynthetic genes, *YUC2* and *YUC6*, in the sporophytic pollen mother cell was essential for the early stages of pollen development (Yao et al., 2018). *yuc2yuc6* is a male sterile double mutant, and the expression of the bacterial auxin biosynthetic gene *iaaM* under the control of the *YUC6* promoter could restore the fertility of *yuc2yuc6*, indicating that the fertility defects of *yuc2yuc6* were caused by partial auxin deficiency during anther development (Cheng et al., 2006). One open question was whether the sporophytic effect comes from the pollen mother cell directly or through the tapetum. To address this question, ectopic production of auxin in the tapetum failed to rescue the sterile phenotype of *yuc2yuc6*. Whereas, production of auxin in either pollen mother cells or microspores rescued the defects of pollen development in *yuc2yuc6* double mutants. This establishes the direct involvement of genetic factors to control the diploid-to-haploid ontogeny switch (Yao et al., 2018).

The important role of auxins in microspore development is further corroborated by several investigations focusing on the characterization of stress-induced microspore embryogenesis (Rodríguez-Sanz et al., 2015; Testillano, 2019). As this process is extremely taxa-specific, understanding the function of auxins during this process will have commercial benefits in accelerating crop breeding and improvement. Taxa-independent establishment of microspore embryogenesis and increased efficiency will improve the production of isogenic doubled haploid lines suitable for breeding purposes (Pérez-Pérez et al., 2019).

In Arabidopsis, *CALLOSE DEFECTIVE MICROSPORE1* (*CDM1*) is another meiotic gene that is essential for the development of microspores (Lu et al., 2014). CDM1 plays an important role in the regulation of callose metabolism which is highly expressed in meiocytes and tapetum and the *cdm1* knockout mutant is male sterile and here also the onset of phenotype starts after the tetrad stage.

All these diverse studies as presented in Figure 2 establish the direct involvement of sporophytic genetic factors in controlling the development and transition of the sporophyte-to-gametophyte stage. In Arabidopsis, *BELL1* expression is high during female gametophyte development; in *bel1*, the female gametophyte fails to develop (Ray et al., 1994; Reiser et al., 1995). It is interesting to note that *BELL1* expression remains low during pollen developmental stages. It will be worth testing the ectopic overexpression of *BELL1* during the pollen developmental stages (Table 2; data obtained from Arabidopsis eFP Browser, Winter et al., 2007).

**TABLE 2:** Tissue-specific expression levels of BELL1 (At5g41410) in Arabidopsis.

Tissue	*AtBELL1* expression level (Absolute)
Cauline leaf	222.3
Cotyledon	109.3
Flower stage 9	33
Flower stage 10/11	70
Mature pollen	24.7

### Haploid-to-Diploid Switch

Genetic control for the haploid-to-diploid switch is not yet thoroughly established. One of the genes that perform this function is *SHORT SUSPENSOR* (*SSP*). *SSP* is an interleukin-1 receptor-associated kinase (IRAK)/Pelle-like kinase gene that is expressed in pollen but remains untranslated until fertilization and thereafter accumulates in the zygote and the endosperm. SSP acts upstream to YODA (YDA) which requires MAPKKK activity for its activation and proper differentiation of the zygote (Lukowitz et al., 2004). SSP protein produced from paternal transcripts upon fertilization triggers zygotic YDA activity (Bayer et al., 2009). This is a classical study to establish how genes expressed in one ontogeny are required to regulate the essential function of another ontogeny.

It is, thus, evident that ORO, SAM, GSM1, GSP1, KNOX2, and BELL1 are molecular regulators that control gametophyte-to-sporophyte phase transitions in *Phaeophyta*, *Chlorophyta*, and *Bryophyta*. However, in angiosperms such studies are still lacking, possibly because, with the increasing occurrence of gene duplications the molecular controls are much more complex. For instance, the number of *BELL* and *KNOX* genes is much higher in dicots and monocots compared with those in the non-vascular plants (Horst et al., 2016; Table 3). Accordingly, the identification of individual functions is hampered by difficulties in generating a loss of function mutants. Moreover, based on different interacting partners at different developmental stages, the same protein performs multiple functions at different developmental stages. This makes it a challenge to decipher specific *BELL* and *KNOX* genes involved in the transition of ontogeny.

**TABLE 3:** Number of *BELL* and KNOTTED1-like (*KNOX*) family proteins across the plant species.

	*Clamydomonas*	*Physcomitrium*	*Arabidopsis*	Poplar	Rice
BELL	1	4	13	15	12
KNOX	1	5	8	19	14

## Translational Applications of Ontogeny Regulation

Microspore embryogenesis is an *in vitro* system in which the haploid microspore is reprogrammed by the application of external stress treatments to enter into the embryogenesis pathway which usually characterizes the diploid sporophytic development and occurs after fertilization (Shariatpanahi et al., 2006). The resulting embryo can be diploidized by the application of chromosome doubling agents, producing doubled haploid (DH) plants. DH plants are important biotechnological tools in plant breeding mainly because they permit the breeding process to be considerably shortened. This is due to the fact that homozygous stable lines are produced within only one generation while this process usually requires at least six generations of backcrossings when traditional breeding is applied (Testillano, 2019). Despite numerous stress applications and chemicals which are known to induce microspore embryogenesis, a better understanding of genes involved in ontogeny transition and regulation would help to engineer the expression of these genes to increase the frequency of microspore embryogenesis. Also, it might help to break the recalcitrance in certain plant species where microspore embryogenesis is not yet successful.

Usually, the embryogenesis event happens from tetrad, microspore, and bicellular pollen stages of the pollen developmental pathway. Stress-induced alterations of the chromatin architecture occurring during these stages to allow access and transcription of key genes is required to skip the pollen development pathway and follow the embryogenesis pathway. A better understanding of ontogeny regulatory genes and their association to the chromatin architecture at different pollen developmental stages might help to engineer and initiate embryogenic pathways at meiotic stages to obtain diploid embryos from the pollen mother cell.

The genetic segregation accompanying meiosis during the diploid-to-haploid switch is causal for the recurrent need to generate seeds for hybrid crops from their homozygous, genetically different parents. Thus, the advantageous heterozygosity of hybrids could be maintained if clonal progenies were generated through seed propagation, e.g., through induced embryogenesis in gametophytes which were generated by mitosis and not by meiosis in corresponding mutants. Recent findings on the capability to induce embryogenesis associated with the transcription factor BABY BOOM1 (BBM1) suggest a possible solution. BBM1 belongs to the superfamily of APETALA 2/ETHYLENE RESPONSE FACTOR (AP2/ERF) transcription factors and was shown to induce somatic embryogenesis when expressed ectopically in several taxa (Boutilier, 2002; Lowe et al., 2016; Méndez-Hernández et al., 2019; Wójcik et al., 2020). In rice gametes, *BBM1* is exclusively expressed in sperm cells but not in egg cells. Moreover, it has recently been found that *BBM1* expression in rice zygotes is specific for the *BBM1* allele introduced with the male gamete, but is expressed biallelic several hours following fertilization (Khanday et al., 2019). Moreover, a triple knockout of *BBM1* along with its two homologs in rice, *BBM2* and *BBM3*, causes embryo arrest and abortion, but can be fully rescued by male-transmitted *BBM1*. These findings suggest that embryogenesis following fertilization requires *BBM1* transmitted from the male genome. Interestingly, this can be applied to induce embryogenesis in egg cells prior to fertilization by male gametes as shown by transgenic rice lines expressing *BBM1* under control of an egg-cell-specific promoter which are parthenogenetic. If *BBM1* is expressed egg-cell-specifically in a genetic background in which meiosis was substituted with mitosis and thus recombination was eliminated (*MiMe* lines; Mieulet et al., 2016), sexual propagation without genetic segregation can be engineered in a sexually reproducing plant. These clonal progenies retain genome-wide parental heterozygosity which is beneficial, e.g., for maintaining hybrids with favorable gene combinations.

Dual ontogeny is an integral part of land plants. A better understanding of ontogeny determinants and controls is not only important for a better understanding of the evolution of plant diversity but will also have commercial benefits. Identifying key genes and altering their expression to switch microspores to reprogram themselves to a diploid ontogeny will be highly beneficial for faster breeding of crop plants, e.g., via the induced production of dihaploids.

## Author Contributions

SP, AT, KP, and RW wrote the manuscript. AM and OD discussed the manuscript. All authors have read the manuscript and approved it for submission.

## Conflict of Interest

The authors declare that the research was conducted in the absence of any commercial or financial relationships that could be construed as a potential conflict of interest.

## Publisher’s Note

All claims expressed in this article are solely those of the authors and do not necessarily represent those of their affiliated organizations, or those of the publisher, the editors and the reviewers. Any product that may be evaluated in this article, or claim that may be made by its manufacturer, is not guaranteed or endorsed by the publisher.
